# Chloroplasts-mediated biosynthesis of nanoscale Au-Ag alloy for 2-butanone assay based on electrochemical sensor

**DOI:** 10.1186/1556-276X-7-475

**Published:** 2012-08-23

**Authors:** Yixia Zhang, Guo Gao, Qirong Qian, Daxiang Cui

**Affiliations:** 1 Department of Bio-Nano-Science and Engineering, National Key Laboratory of Nano/Micro Fabrication Technology, Key Laboratory for Thin Film and Microfabrication of Ministry of Education, Institute of Micro-Nano Science and Technology, Shanghai Jiao Tong University, 800 Dongchuan Road, Shanghai, 200240, People’s Republic of China; 2 Department of Orthopaedics, Changzheng Hospital affiliated to Second Military Medical University, 451 Fengyang Road, Shanghai, 20003, People’s Republic of China

**Keywords:** Chloroplasts, Au-Ag alloy, Nanosensing film

## Abstract

We reported a one-pot, environmentally friendly method for biosynthesizing nanoscale Au-Ag alloy using chloroplasts as reducers and stabilizers. The prepared nanoscale Au-Ag alloy was characterized by UV–visible spectroscopy, X-ray diffraction (XRD) and high resolution transmission electron microscopy (HR-TEM). Fourier transform infrared spectroscopy (FTIR) analysis was further used to identify the possible biomolecules from chloroplasts that are responsible for the formation and stabilization of Au-Ag alloy. The FTIR results showed that chloroplast proteins bound to the nanoscale Au-Ag alloy through free amino groups. The bimetallic Au-Ag nanoparticles have only one plasmon band, indicating the formation of an alloy structure. HR-TEM images showed that the prepared Au-Ag alloy was spherical and 15 to 20 nm in diameter. The high crystallinity of the Au-Ag alloy was confirmed by SAED and XRD patterns. The prepared Au-Ag alloy was dispersed into multiwalled carbon nanotubes (MWNTs) to form a nanosensing film. The nanosensing film exhibited high electrocatalytic activity for 2-butanone oxidation at room temperature. The anodic peak current (Ip) has a linear relationship with the concentrations of 2-butanone over the range of 0.01% to 0.075% (v/v), when analyzed by cyclic voltammetry. The excellent electronic catalytic characteristics might be attributed to the synergistic electron transfer effects of Au-Ag alloy and MWNTs. It can reasonably be expected that this electrochemical biosensor provided a promising platform for developing a breath sensor to screen and pre-warn of early cancer, especially gastric cancer.

## Background

Bimetallic nanoparticles display different catalytic properties, surface energy and magnetic properties depending on the different types of metal nanoparticles [1-4]. Au–Ag bimetallic nanoparticles attract great attention due to their unique optical, electrochemical properties and important applications as biosensors [5-8]. Integration of ‘green chemistry’ and ‘sustainable processes’ principles into nanotechnology is important for designing environmentally friendly nanomaterials. Recently, microorganisms and extracts of plants have been used for synthesizing Au-Ag bimetallic nanoparticles [9-12]. The above biological methods were clean, eco-friendly and safe. However, they involved tedious work, such as medium culture, long experiment procedure and complicated process. Biosynthesis of bimetallic Au-Ag nanoparticles using chloroplasts can potentially eliminate these problems. Chloroplasts are abundant in green plants and can be conveniently isolated. No tedious medium culture work is required and the experiment can be easily scaled up for large-scale synthesis. We have successfully prepared gold nanoparticles using chloroplasts in our early work [13]. In this paper, we report the biosynthesis of nanoscale Au-Ag alloy in chloroplasts solution and further test the possibility to use Au-Ag alloy as an electrochemical biosensor.

Electrochemical biosensors have been one of the powerful tools for cancer diagnostics, as a simple-preparation, low-cost, rapid-response and portable platform [14-16]. The coupling of electrochemical devices with nanomaterials offers a unique capability for accurate measurements of multiple cancer markers from a variety of cancer patient samples. Au-containing bimetallic nanoparticles have been expected to enhance the catalytic activity and selectivity of electrochemical sensors [17-20].

Breath analysis has been proposed as a convenient, noninvasive and safe method for detecting early stage cancer, in comparison with the traditional diagnostic techniques [21,22]. Up to now, breath analysis was done by GC/MS or PTR/MS [23-25]. However, the high-cost, time-consuming sampling procedure and tedious data analysis of MS technology limited their clinical applications. Much less work had been devoted to analyze volatile biomarkers in exhaled breath of cancer patients based on electrochemical biosensors since, 2-butanone was identified as a volatile indicator in the breath of gastric cancer [26,27]. We investigated the electrocatalytic properties of the Au-Ag/MWNT nanosensing film for low concentration of 2-butanone.

## Methods

### Reagents

Chloroauric acid tetrahydrate (HAuCl_4_·4H_2_O), sodium chloride (NaCl), potassium chloride (KCl), sodium hydroxide (NaOH), and silver nitrate (AgNO_3_) were purchased from Sigma-Aldrich (St Louis, MO, USA). All reagents were of commercial grade and used without further purification. Water used in experiments was ultrapure grade. Trifolium were collected from the campus of Shanghai Jiao Tong University. Shanghai, People’s Republic of China. Acid-treated MWNTs were supported by Qifa Liu PhD, coming from Shanghai Jiao Tong University.

### Apparatus

Homogenizer (PRO200, Ginotech, Shanghai, People's Republic of China), UV spectrophotometer (Cary 50, Varian, Shimadzu, Tokyo, Japan), HR-TEM (JEOL-2100F, JEOL Ltd., Tokyo, Japan), INCA energy spectrometer (Oxford Instruments, Abingdon, UK), X-ray diffractometer (D/max-RC, Rigaku Corporation, Tokyo, Japan), Fourier transform infrared spectrometer (EQUINOX 55, Bruker Optics, Bruck, Germany), and electrochemical workstation (Model CHI660D, CH Instruments, Austin, TX, USA) were used.

### Biosynthesis and characterization of nanoscale Au-Ag alloy

The chloroplast stock suspension was obtained by the same method reported in our previous work [13]. The pH value of the HAuCl_4_ solution was adjusted to around 7.5 by adding 1 M NaOH solution. Typically, in preparing Au-Ag alloy, 30 mL vial was added to 2 mL of 10-mM HAuCl_4_ solution and 5-mL chloroplast solution, followed by the addition of 2 mL of 10-mM AgNO_3_ solution to the reaction mixture. The final volume of the reaction solution was adjusted to 20 mL using deinoized water and stirred continuously at 25°C for 16 h. The color changes of the reaction solution were observed from light green to brown and finally to dark purple, which indicated the formation of Au-Ag alloy.

The reaction solution was subjected to UV-visible (UV-vis) absorption measurements. UV-vis spectra were recorded as a function of reaction time on a UV spectrophotometer, operating at a resolution of 1 nm. The spectra were collected over the range of 200 to approximately 800 nm with a scanning speed of 1,836 nm/min. Samples for high-resolution transmission electron microscopy (HR-TEM) analysis were prepared by drop-coating reaction solutions onto carbon-coated copper, followed by drying prior to HR-TEM measurements. Powder X-ray diffraction (XRD) patterns of the Au-Ag alloy were collected on a D/max-RC X-ray diffractometer operating at a voltage of 45 kV and a current of 40 mA with Cu-Kα1 radiation at *λ* = 1.540 Å in the 2*θ* range from 20° to 90°. For Fourier transform infrared (FTIR) spectroscopy measurements, the reaction solution was centrifuged at 10,000 rpm for 15 min; following with the pellet re-dispersed in distilled water to get rid of any free biological molecules. The process of centrifugation and re-dispersion in distilled water was repeated three times to ensure better separation of free entities from the alloy. The purified pellets were freeze-dried, and the powders were subjected to FTIR spectroscopy measurements. The FTIR measurements were carried out on an instrument in the diffuse reflectance mode operating at a resolution of 4 cm^−1^ over 4,000 to approximately 400 cm^−1^. Meanwhile, acid-treated MWNTs and chloroplasts were for FTIR analysis under the same condition as Au-Ag alloy

### Electrochemical assay

For the preparation of Au-Ag/MWNT-modified electrode, the glass carbon electrodes were polished with 0.3 and 0.05 μm alumina powders on a micro-cloth, respectively. Then, electrodes were thoroughly cleaned ultrasonically with ethanol and ultrapure water. After that, they were dried under N_2_ blow. Typically, 20 μL of 10-mM suspension of Au-Ag was dispersed into 20 μL of 0.1-mg/mL MWNT water solution for ultrasound for 5 min. Au-Ag/MWNT mix solution (10 μL) was dropped onto the surface of glass carbon electrode and left it at ambient conditions to dry.

Cyclic voltammetry (CV) measurements were carried out with electrochemical workstation. A conventional three-electrode system was composed of a modified glass carbon electrode as working electrode, a platinum wire auxiliary electrode, and a saturated calomel electrode (SCE) as the reference electrode. Three electrodes were inserted into the 20-mL deaerated KCl solution for electrochemical assay. Prior to CV measurements, the working electrode was swept from −0.5 to 0.5 V (pH 7.4) until the response was stable. The experimental conditions, such as supporting electrolyte and scan rate, as well as the ratio between MWNTs and Au-Ag have been optimized.

## Results and discussion

### Characterizations of Au-Ag alloy nanoparticles

**Figure 1:**
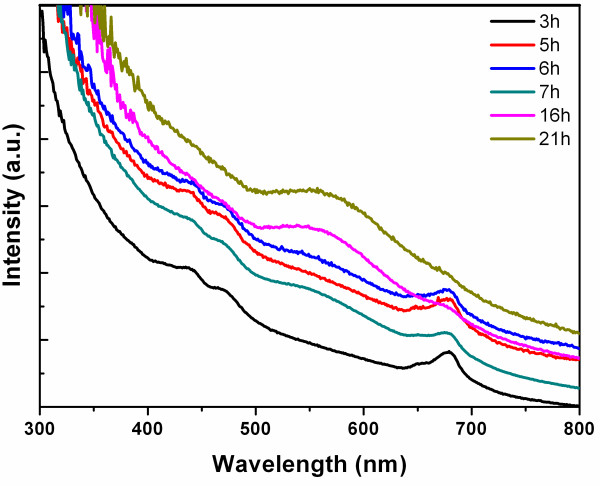
**UV-vis spectra.** The UV-vis spectra were recorded as a function of reaction time in the range of 300 to 800 nm, when equimolar ratio of HAuCl_4_ and AgNO_3_ were introduced into the chloroplast solution. The time intervals are 3, 5, 6, 7, 16, and 21 h.

Equimolar HAuCl_4_ and AgNO_3_ solutions were introduced into the chloroplast solution for preparing nanoscale Au-Ag alloy. After 16 h, the color of the reaction solution gradually changed from light green to brown, then to dark purple. This clearly suggests the formation of nanoscale Au-Ag alloy. UV-vis spectra were recorded as a function of time from 3 to 21 h (Figure 1). It was obvious that the nanostructure Au-Ag alloy began to appear at 7 h, and the reaction almost completed at 16 h. The presence of only one plasmon resonance band in UV-vis absorption at 548 nm indicated that the nanostructure Au-Ag was formed as an alloy [28,29] rather than either a segregated metal or a core/shell structure characterized by two SPR bands [5,6]. 

**Figure 2:**
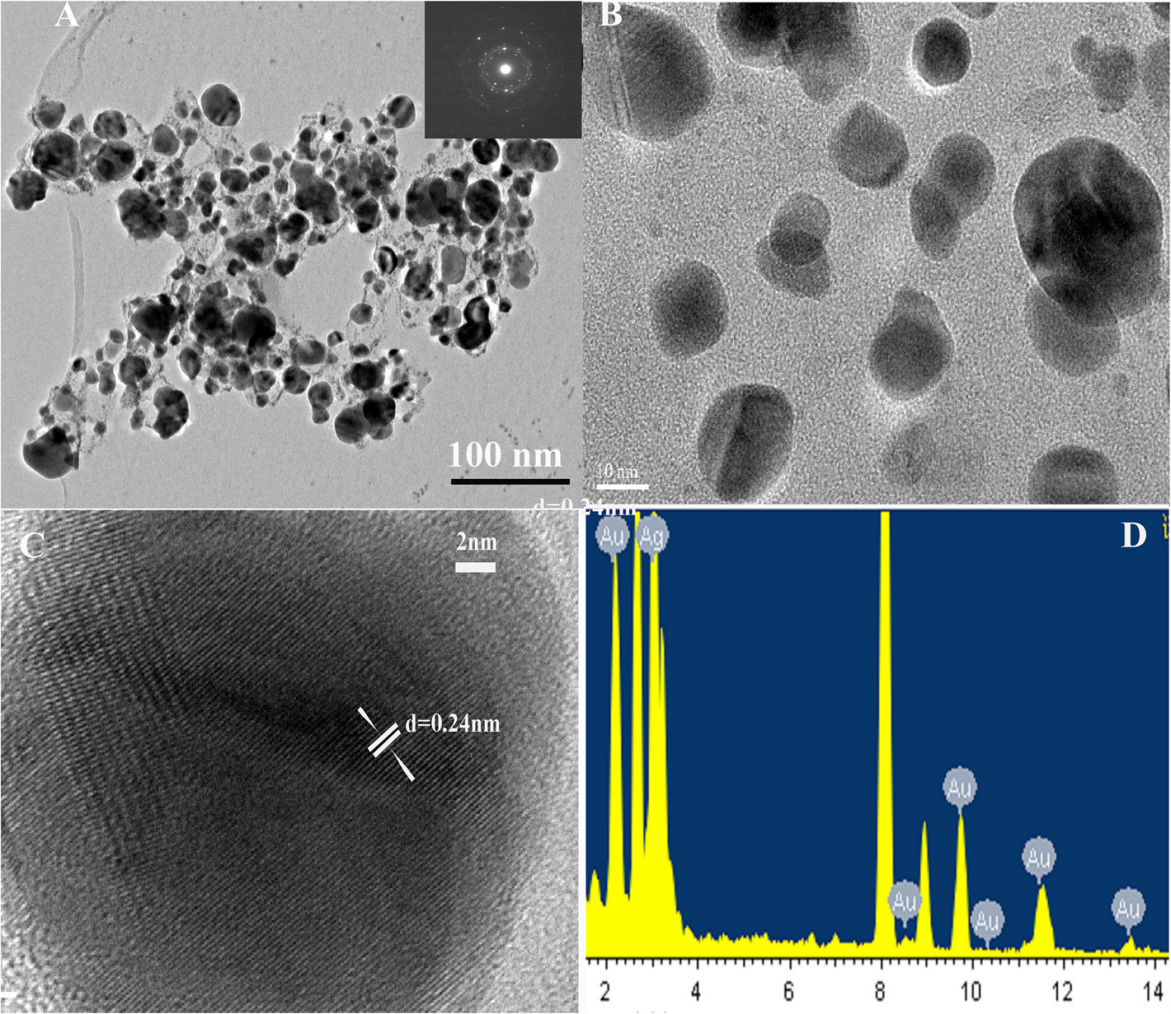
**TEM and HR-TEM images.** (**A**) TEM images of Au-Ag alloy: the inset was the corresponding SAED patterns, (**B**) high-magnification TEM images, (**C**) HR-TEM image of single Au-Ag alloy particle, and (**D**) energy dispersive X-ray spectroscopy measurement profiles.

The size and microstructure of the prepared Au-Ag alloy were further examined by HR-TEM micrographs (Figure 2A). TEM imaging displayed that the Au-Ag alloy was spherical with diameters of 10 to 20 nm. Four typical electron diffraction patterns for the Au-Ag alloy were identified (the inset in Figure 2A), in which the radii of the four main fringe patterns were all in the ratio of $\sqrt{3}$: 2: $\sqrt{8}$: $\sqrt{11}$. They were related to the (111), (200), (220), and (311) planes, which confirmed the face-centered cubic (fcc) structure of the Au-Ag alloy. The crystallinity and crystal structure of Au-Ag alloy were examined by HR-TEM and XRD analysis. The HR-TEM assay result showed that the as-prepared nanocomposites own the typical nanostructure of the Au-Ag alloy (Figure 2C) and was with the presence of local defect structures; the individual lattice planes were clearly visible. Spot-profile energy dispersive X-ray (EDX) analysis of a number of prepared nanoparticles constantly confirmed the presence of both Au and Ag signals (Figure 2D).

**Figure 3:**
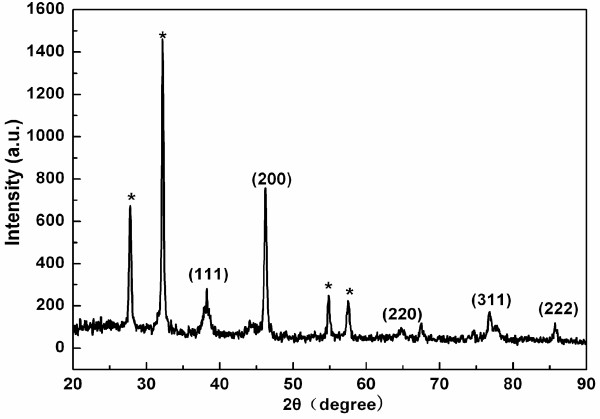
**XRD patterns of Au-Ag alloy synthesized by chloroplasts with AgNO**_**3**_** and HAuCl**_**4**_** aqueous solutions.** Peaks marked with stars arising from the crystalline bioorganic phase.

The XRD pattern of the nanoscale Au-Ag alloy was shown in Figure 3. Five characteristic peaks (111), (200), (220), (311), and (222) confirmed that the Au-Ag alloy was in the fcc structure [13,30]. The peak corresponding to the {200} plane was more intense than the other planes, suggesting that the {200} plane was the predominant orientation, which demonstrated that the nanoscale Au-Ag alloys are high crystalline [7,31]. 

**Figure 4:**
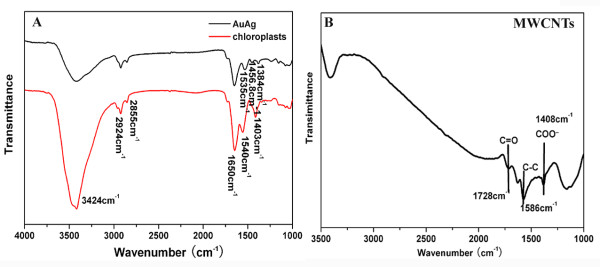
**(A) FTIR spectra of chloroplasts and Au-Ag alloy scanning at a range of 4,000 to approximately 1,000 cm**^−1^**; (B) FTIR spectra of acid-treated MWNTs.**

FTIR analysis was proceeded to verify the responding biomolecules from the chloroplasts acting as reducers and stabilizers for bio-reduction of the nanoscale Au-Ag alloy (Figure 4). The intense band centered at 3,424 cm^−1^, both in chloroplasts and Au-Ag alloy, was due to stretching and bending vibrations in combination involving OH and NH groups (Figure 4A) [32]. Two peaks occurring at 2,924 and 2,855 cm^−1^ were assigned to methylene (sym/antisym) vibrations of the hydrocarbons present in proteins or enzymes [33,34]. The 1,650-cm^−1^ prominent band was attributed to amide I vibrations corresponding to stretching of carbonyl groups in amide linkages [35]. The peak 1,535 cm^−1^ was considered as the N-H stretching modes of vibration in the amide II linkages of polypeptides/proteins [36]. The 1,403-cm^−1^ band arising from the chloroplasts could be assigned to the COO^−^ symmetric stretch from carboxyl side groups in the amino acid residues [37]. The disappearance of band at 1,403 cm^−1^ clearly demonstrated that biomolecules with carboxyl groups acted as actors for co-reduction of Ag^+^ and Au^+^. The FTIR band at 1,384 cm^−1^ coming from Au-Ag alloy corresponded to C=C stretching of aromatic amine group or C-N stretching vibrations of aromatic amines [38]. FTIR profiles suggested that proteins were bound to the surface of Au-Ag alloy nanoparticles through amine groups, which stabilized the alloy. The peak shifts from 1,540 to 1,535 cm^−1^, which indicate that proteins play a role as reducers for Au-Ag alloy. The results demonstrated that proteins play as a role of capping and reducing agents for co-reducing ions of Au and Ag. According to Shakibaie's work [39], these chemical groups could be used to conjugate with other functional groups for special applications. 

Figure 4B had shown the FTIR spectrum of acid-treated MWNTs. The absorption band at 1,586 cm^−1^ is assigned to the C-C stretching vibration of the MWNT backbones. The weak absorption band located at 1,728 cm^−1^ is assigned to the carbonyl stretching vibration. The 1,408-cm^−1^ band was attributed to the vibration of COO^−^[40]. The results indicate that carboxylic groups and carboxyl groups have been successfully introduced onto the MWNT backbones after oxidation with concentrated HNO_3_. These chemical groups are beneficial to the Au-Ag alloy covalent interaction with MWNTs, forming amide linkages between amine residues and carboxylic acid groups.

### Electrochemical analysis

**Figure 5:**
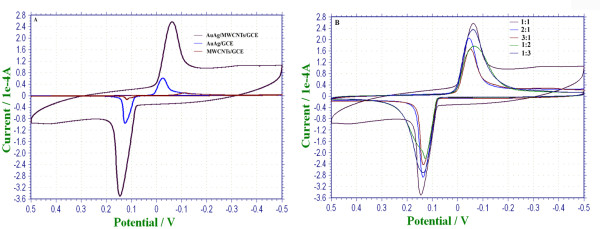
**The amperometric response of Au-Ag/MWNT working electrode.** (**A**) Curves of CV were obtained with different working electrodes (MWNTs/GCE, Au-Ag/GCE, and Au-Ag/MWNTs/GCE); (**B**) the amperometric response of Au-Ag/MWNT working electrode with different molar ratios between Au-Ag and MWNTs (3:1, 2:1, 1:1, 1:2, and 1:3, respectively).

According to experimental results, 0.1-mol/L KCl solutions was the buffer solution with the highest current response and the best stability of working electrodes, compared with PBS, NaNO_3_, and NaCl solutions. Therefore, 0.1-mol/L KCl solution was chosen to evaluate the electrocatalytic properties of the Au-Ag/MWNT nanocomposite film. Cyclic voltammograms of different working electrodes were performed at potential ranges from −0.5 to approximately 0.5 V (vs. SCE), as shown in Figure 5A. Meanwhile, the properties of nanocomposite film, composed of different ratios of Au-Ag and MWNTs, were evaluated, with the ratio of 3:1, 2:1, 1:1, 1:2, and 1:3, respectively (Figure 5B).

By comparing the amperometric response of different working electrodes (MWNTs/GCE, Au-Ag/GCE, and Au-Ag/MWNTs/GCE), a pair of well-defined redox peaks and broader response window were obtained through Au-Ag/MWNTs/GCE. The peak current of Au-Ag/MWNTs/GCE (*E*_p_ = −0.062 V, *I*_p_ = 220.3 μA) is 4.2 times greater than the Au-Ag/GCE (*E*_p_ = −0.031 V, *I*_p_ = 51.56 μA) peak current. Moreover, the peak potential exhibited a negative shift of 31 mV (vs. SCE). The experiment has been repeated for five times, and the results are the same. The strong current response of Au-Ag/MWNTs/GCE might be due to the synergetic electron transmission of both Au-Ag and MWNTs. According to the work of Wang et al. [41], Au-Ag alloy has strong synergistic effects, which increase the catalytic activities for CO oxidation, compared to Au or Ag alone. An interesting result was observed that the ratios of components in the nanofilm affect the sensitivity of the electrochemical response (Figure 5B). When the ratio between Au-Ag alloy and MWNTs was 1:1, the highest current response of the nanofilm was obtained. Thus, the molar ratio of 1:1 between Au-Ag alloy and MWNTs was chosen to detect 2-butanone in this paper.

**Figure 6:**
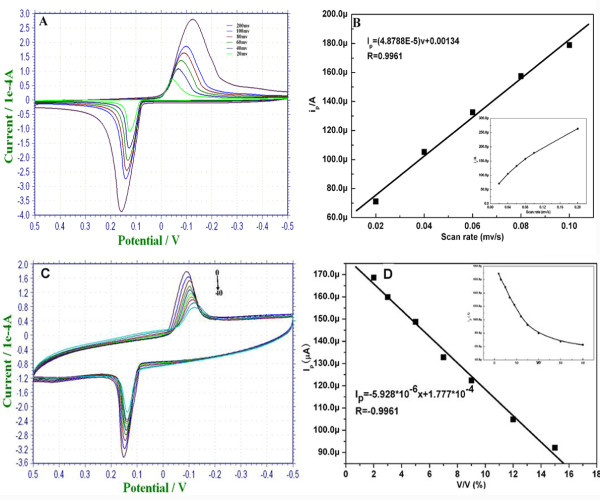
**The performance of cyclic voltammograms.** (**A**) Cyclic voltammograms of the Au-Ag/MWNT working electrode at various scan rates from 20 to 200 mV/s in 0.1-mol/L KCl solution. All potentials are given versus SCE; (**B**) displays the dependence of the redox peak currents (*I*_p_) on the scan rates (*v*). The inset shows redox peak currents plot with scan rate at a range of 20 to approximately 200 mV/s. (**C**) Cyclic voltammograms of Au-Ag/MWNTs/GCE were exposed to various concentrations of 2-butanone at ranges of 0% to approximately 20% (*v*/*v*) in 0.1-M KCl solution; (**D**) the linear calibration plot of the peak current against butanone. The inset shows the curve of the peak current corresponding to different concentrations of 2-butanone.

In order to investigate the rate-limiting step of 2-butanone electrochemical oxidation on Au-Ag/MWNTs/GCE, the effects of scan rate on the oxidation peak current was recorded at potential ranges of −0.5 to 0.5 V (vs. SCE) with different scan rates in 0.1-mol/L KCl solution (Figure 6). The results displayed that the anodic peak current increased with a negative shift of potential when the scan rates varied from 20 to 200 mV/s. The anodic peak current versus the sweep rate plot was shown in Figure 6B. There was a good linear relationship between the current of oxidation peak (*I*_p_) and the scan rate (*v*) at a range of 20 to 100 mV (Figure 6B). The equation is: *I*_p_ = 4.8788 × 10^−5^ V + 0.000134 (*R* = 0.9961). The peak current was directly proportional to the scan rate, which indicates that the reaction of electrochemical catalytic oxidation of 2-butanone on Au-Ag/MWNTs/GCE was an adsorption-control process.

The electrocatalytic ability of the nanosensing film toward 2-butanone was investigated in 0.1-M KCl solution under room temperature. It was observed that the peak current descended with the increase of 2-butanone concentration along with negative shift of peak potential (Figure 6C). A good linear relationship between the oxidation peak current *I*_p_ and concentrations of 2-butanone (*x*) was obtained at ranges of 0.01% to 0.075% (*v*/*v*) (Figure 6D). The calibration curve equation was *I*_p_ = −5.928 ×10^−6^*x* + 1.777 ×10^−4^ with *R* = −0.9961.

### Reproducibility and stability

The reproducibility of the electrochemical sensor was examined by analysis of the same concentration of 2-butanone (15 μL) using five equally prepared electrodes. The results showed that the current responses were 92.21, 95.36, 90.79, 96.23, and 97.27 μA, respectively. It was confirmed that the electrochemical sensor had good reproducibility with a relative standard deviation of 3.78% (less than 5.0%).

We also examined the storage stability of the developed electrochemical sensor. The electrochemical sensor retained 90.4% of its initial response, when stored in KCl solution for 1 week. The slight decrease in current response may be attributed to electron transfer between Au-Ag alloy and MWNTs.

## Conclusions

One simple and eco-friendly way to synthesize nanoscale Au-Ag alloy has been successfully developed using chloroplasts as the reducers and stabilizers at room temperature. The Au-Ag alloy was dispersed into MWNTs, as a sensing film, to modify glass carbon electrodes. The modified electrode was subjected to assay 2-butanone using CV. This transducer-free and membrane-free electrochemical sensor exhibits fast response, good reproducibility, sensitivity, and stability for the detection of 2-butanone at room temperature; its linear range was obtained at ranges of 0.01% to approximately 0.075% (*v*/*v*).

The electrochemical biosensor based on nanocomposites of Au-Ag/MWNTs could be further used for the detection of other volatile biomarkers from the breath or oral gas of gastric cancer patient. It has great potential in screening and pre-warning early gastric cancer in the near future.

## Competing interests

The authors declare that they have no competing interests.

## Authors' contributions

YZ carried out the preparation of nanoscale Au-Ag, electrochemical test and drafted the manuscript. GG participated in the design of the study and performed the statistical analysis. QQ participated in the sequence alignment. DC conceived of the study and participated in its design and coordination. All authors read and approved the final manuscript.
